# Determinants of FDI Localization in China: A County-Level Analysis for the Pharmaceutical Industry

**DOI:** 10.3390/ijerph14090985

**Published:** 2017-08-30

**Authors:** Su Li, Antonio Angelino, Haitao Yin, Francesca Spigarelli

**Affiliations:** 1 Antai College of Economics and Management, Shanghai Jiao Tong University, 1954 Huashan Road, Shanghai 200030, China; lisu_sjtu@sjtu.edu.cn; 2 Department of Economics and Management, University of Ferrara, Via Voltapaletto 11, 44121 Ferrara, Italy; nglntn@unife.it; 3 China Center, University of Macerata, Via Piaggia dell’Università 2, 62100 Macerata, Italy; francesca.spigarelli@unimc.it

**Keywords:** FDI location, China, pharmaceutical industry

## Abstract

Foreign direct investments (FDIs) have been widely recognized as a crucial feature of the Chinese industrial development process. Over the past decades, China has been attracting huge amounts of inward FDIs as a consequence of both spontaneous market dynamics and place-based preferential policies at the sub-national level. However, the Chinese market exhibits large dissimilarities in terms of FDI localization across territories that are worth investigating at a more disaggregated level. In this regards, our study explores the determinants of attraction of inward FDIs in China, at the county level. It focuses on the pharmaceutical industry and attempts to assess whether factors related to location advantages, agglomeration dynamics, information cost effects and environmental regulation costs affect foreign firms’ localization choices as well as invested amounts in that location. By means of discrete choice models, our paper confirms the findings of the prevalent literature about the positive effects of location advantages on pharmaceutical FDI attraction. Different from our expectations, a higher proportion of foreign enterprises do not stimulate significant effects on FDI localization, while preferential policies and sectoral agglomeration are positively correlated with the localization of pharmaceutical foreign firms. Finally, our results suggest that investing firms tend to avoid areas with strict environment regulation.

## 1. Introduction

In the course of the past few decades, China has been experiencing rapid economic development. Foreign direct investment as a source of capital, knowledge transfer and export competitiveness has been playing an important role in the Chinese industrialization process [1]. Since the beginning of the Nineties, foreign investments have significantly affected the structural transformation of the Chinese economy, contributing to the country’s progressive specialization towards higher value added sectors [2].

**Figure A1:**
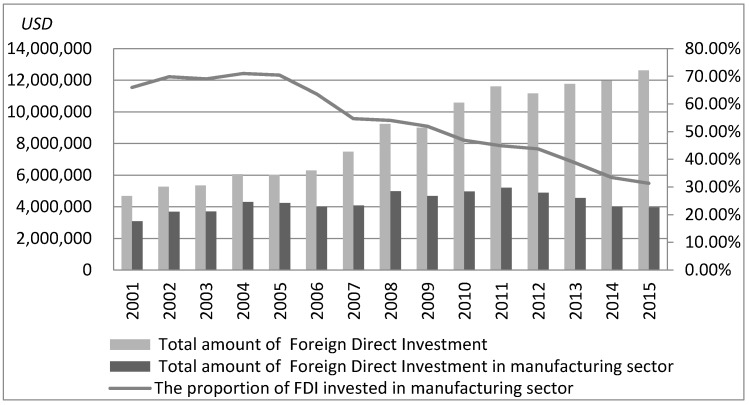
Foreign Direct Investment in China (2001–2015). Source: China Macroeconomic Database.

The total amount of Foreign Direct Investment (FDIs) tripled during the past 15 years in China, increasing from 46.9 billion U.S. dollars in 2001 to 126.2 billion U.S. dollars in 2013, with the largest proportion based in the manufacturing sector (Figure A1 in Appendix A).

**Figure A2:**
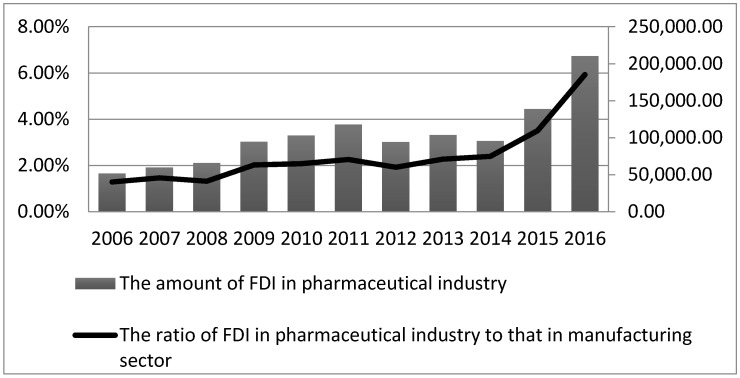
FDI in Pharmaceutical Industry (2006–2016). Source: China’s Macroeconomic Database.

In this research, we investigate the dynamics of FDI inflows in the Chinese pharmaceutical industry. From an economic perspective, the pharmaceutical industry in China has experienced a rapid growth, mainly from the mid 2000s [3], and an increase of its weight over the total amount of manufacturing FDI [4]. The total value of FDI in the pharmaceutical industry has moved from 0.5 billion U.S. dollars in 2006 to 2.1 billion U.S. dollars in 2016, and the ratio of FDI in the pharmaceutical industry to that in the manufacturing sector has tripled, from 1.29 to 5.93% (Figure A2 in Appendix A). From a policy perspective, the pharmaceutical industry has been regarded as one of the key industries in China’s Tenth Five-Year Plan, Eleventh Five-Year Plan and Twelfth Five-Year Plan, resulting in becoming a target for a series of national industrial policies directed at the expansion and upgrading of its productive capacity [5,6,7,8]. In addition, several provinces and cities have implemented special measures to support development in the pharmaceutical industry by means of preferential policies directed to FDI attraction [9].

In this regards, taking into account the heterogeneity of the Chinese market in terms of territorial assets and policies, our paper intends to assess the determinants of pharmaceutical FDI localization [10,11]. Specifically, we investigate the factors triggering foreign firms’ localization choices at the county level as well as their invested amounts, taking into account the interval 2004–2013. Among the factors explored, we selected four categories of localization determinants—location advantages, agglomeration effects, information cost effects and environment effects. We augment the standard framework of the eclectic paradigm by Dunning [12]—also known as the Ownership Location Advantage (OLI) model—emphasizing the role of ownership, location and internalization advantages in the context of multinational enterprises’ overseas investment decisions.

We intend to contribute to the literature on FDI localization in China [13] by providing new evidence on the determinants of firms’ choices, focusing on a fast growing high value-added and research-intensive industry—pharmaceuticals—and placing emphasis on the territorial level, analyzing the data at a quite disaggregated level—the counties. At the same time, our analysis employs a wide set of variables to capture the impact of tangible and intangible territorial assets as well as institutional and political variables on the localization of pharmaceutical FDIs’.

In terms of policy implication, a more precise understanding of the key factors affecting FDI localization is crucial for the government’s formulation of relevant place-based specialization policies directed at enhancing international competitiveness as well as at mitigating territorial differences. Besides, our analysis provides some insights on FDI localization dynamics and on possible attraction policies with respect to other emerging countries in the East Asian region.

The paper is structured as follows. Section 2 briefly reviews the theoretical foundations for location determinants for our analysis starting from the OLI model and enriching it with other determinants of localization recognized by the literature. Section 3 is divided in two subsections. The first provides a geographical mapping of pharmaceutical FDIs’ localization trends across counties. The second part describes the variables adopted in our model and the empirical methods used for the analysis. Section 4 exhibits the main results of the regressions. Finally, several concluding remarks are contained in Section 5.

## 2. Literature Review

Over the last few decades, the academic literature has devoted significant attention to the issue of FDI location determinants. In particular, the regional determinants of inward FDI distribution have been developed and explained from different perspectives, considering the territorial differences in terms of tangible and intangible assets, policy incentives and investment opportunities as well as focusing on enterprises’ motivations and the business strategy [14,15,16,17]. Nevertheless, despite the large number of theoretical and empirical studies on the location determinants of FDI, it still appears difficult to achieve a unified conclusion. When analyzing the location determinants of FDI, the Eclectic Paradigm of International Production proposed by Dunning is one of the most commonly used theories [18].

In the OLI paradigm, Dunning [18,19,20] emphasizes the importance of ownership advantages, location advantages and internalization for multinational enterprises’ overseas investment decisions. Among these factors, Dunning particularly stresses the role of location advantages as the most critical determinant of inward FDI investments [19].

Following a similar approach, in the presence of location advantages, a foreign firm would be more likely to relocate its production abroad, as directly investing in the host country would result in more cost-saving than exporting overseas. Dunning [18] categorizes location advantages into four factors: (a) market factors including market size, market growth, the degree of proximity with customers and the existing market layout; (b) trade barriers, related to the trade tariffs and local customers preferences; (c) cost factors, consisting of the proximity to supply sources, labor costs, raw material costs, and transportation costs; (d) investment conditions concerning the local political and normative framework in terms of FDI regulations and political stability. From this perspective, the existence of location advantages is a funding condition for foreign direct investment.

Starting from this framework, some of the latest research has integrated the original Dunning’s paradigm including further determinants of FDI location, such as the agglomeration effect, the information cost effect and the environment regulation effect.

With the rise of new economic geography, the agglomeration effect has received increasing attention as a source of FDI location. The economic rationale for agglomeration dynamics derives from Marshall who argued that the spatial concentration of enterprises from the same industry generates positive externalities by means of a cost reduction provided by shared labor markets, professional services and fundamental infrastructure [21]. Porter indicates that industrial correlation and agglomeration are likely to raise firms’ productivity, innovation, performance and industrial competitiveness [22]. In this regards, Dunning highlights the relevance of the spatial agglomeration advantage as an additional driver of multinationals’ investment location [12]. This is even more relevant when Multinational Corporations (MNCs) decide to operate in fragmented markets characterized by higher uncertainty and business risk.

According to the conclusions of a series of empirical studies, foreign enterprises will obtain a wide range of benefits when entering an industrial agglomerated area. Firstly, as implied by He and Liu [23], and Kinoshita and Mody [24], foreign enterprises can deeply understand the host country’s markets through the information provided by previous foreign investors, mainly concerning labor market functioning, modes of cooperation with local agents and the expansion of the economic scale in the host market. Secondly, Belderbos and Carree [25] and Yeung, Liu, and Dicken [26] stress that in the agglomerated area foreign enterprises are more likely to make full use of their internalization advantages acting as both purchasers and suppliers, thus reducing their production costs and business risks in the market. Thirdly, Head and Ries [27] indicate that newly entered foreign enterprises in agglomerated areas can share fundamental infrastructure and social networks with the enterprises already operating in the markets, including skills training, logistics and government services.

In addition to location advantages and agglomeration effects, FDI location choices also seem to be influenced by a series of political and institutional factors. Indeed, compared with domestic enterprises, foreign firms are not familiar with the local business environment, thus facing potential transaction costs linked to the unpredictability of the host country institutional systems that lead them to take on the burden of higher information costs [28]. In their research, Mariotti and Piscitello [29] identify a significant negative relationship between information cost and FDI location across Italian regions. According to their construction, foreign investors collect information through two channels. The first one consists of public information concerning market size, fundamental infrastructure and related policies—this is easy to obtain but not sufficient for a foreign firm to make an overseas investment decision. The other channel consists of learning from previous investment experiences in the host country [30,31,32,33]. In this respect, Mariotti and Piscitello [29] stress the impact of low information cost on FDI localization by proving the positive effects of factors including the distance from the economic center, the previous investment of large multinationals and the age of first foreign investment. Following a similar approach, Kogut and Chang [34] show how Japanese companies tended to adopt “follow-up” strategies in their direct investment in the United States, emphasizing the importance of previous direct investment. Finally, focusing on the Chinese market, He and Liu [23] show that the presence of previous foreign investments in the Beijing area contributed to information spillover effects and lead to industrial concentration of foreign investment.

A last aspect is related to regulation effects on the selection of FDI location, in terms of administrative, labor and environmental standards. The literature has devoted much attention to the environmental rules in China: since the 1980s, the impact of environmental control on the location choice of enterprise has become a central issue in the academic research even though they have not reached a unified conclusion. On one side, there are those who consider the influence of environment regulation intensity as less significant than the agglomeration and the information cost effects [35]. On the other side, some studies imply that foreign enterprises tend to be more sensitive to environment regulation and will locate in areas with less intensive environmental constraints [36,37]. In this regard it is worth highlighting that, due to the lack of information, local governments generally tend to apply more stringent environment regulation policies to foreign enterprises, thus enhancing the administrative burdens on their activities [38]. In an empirical analysis on the determinants of FDI localization in the United States, List and Co [39] found that foreign firms are less likely to invest in places with a higher environmental regulation intensity. On the other hand, data provide different evidence with respect to the Chinese context, where the presence of stringent environmental regulations seem to be associated with a higher probability of attracting foreign investments [39].

## 3. Data and Methodology

### 3.1. Database and Localization of FDIs in China

**Figure 1:**
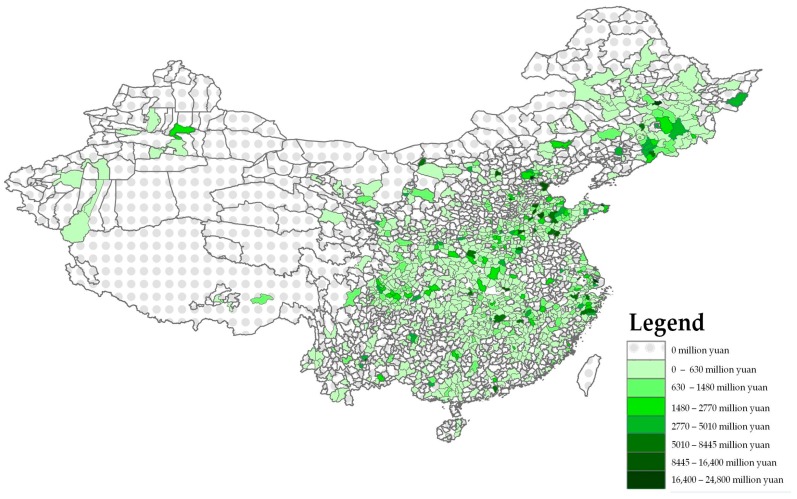
Spatial distribution of total assets of foreign pharmaceutical enterprises in 2013. Source: Figure created by the Authors derived from Arcgis.

Our study makes use of several databases (Table A1). In order to investigate the FDI localization dynamics in the pharmaceutical sector, we use China’s Industrial Database, which collects firm-level data obtained from the “Statistical Report on Scale Statistics” conducted by the National Bureau of Statistics of China over the 2004–2013 period. We aggregate the information related to foreign pharmaceutical firms’ total assets at the county-level. In addition, we use data and information from the China Statistical Yearbook, the government website and the software Arcgis. As mentioned before, the spatial distribution of pharmaceutical FDI across Chinese territories is quite heterogeneous, considering both the stock of total assets and the capital accumulation trends. Such a dynamic appears evident in Figure 1, representing the pharmaceutical FDI intensity in terms of total assets at the county level with respect to the year 2013. As it is possible to see from the map, most of the foreign pharmaceutical enterprises are located in the Eastern areas. In this regards, the first counties in terms of FDI pharmaceutical assets localization are: Huancui District, Yuhua District, Daxing District, Pudong New Area, Daoli District (Table A2); on the other hand, many counties located in the Northern, Central and Western areas exhibit lower or no FDI in pharmaceuticals.

Focusing on the changes that occurred in pharmaceutical FDI total assets over the period from 2004–2013, Figure 2 shows a similar heterogeneous spatial pattern, partially confirming the trends analysed previously. In particular, the map displays significant increases in pharmaceutical FDI total assets have mainly occurred in the Eastern coastal counties as well as in many counties located in the central provinces of Hubei, Hunan and Jianxi (Table A3). At the same time, some other East and Southeast counties located in the Hebei, Liaoning, Jiangsu and Guangdong provinces have experienced a significant reduction in pharmaceutical FDI total assets.

From this perspective, this descriptive analysis of the spatial distribution of pharmaceutical FDI demonstrates that the counties located in the eastern side of the country are likely to be characterized by a higher degree of attractiveness. This may be partially due to their higher performance in terms of Gross Domestic Product (GDP) growth, industrial development and market competitiveness. However, there are many other factors that are likely to influence FDI localization trends. From this perspective, in the following sessions we develop a model to explore the determinants of pharmaceutical FDI localization across Chinese counties.

### 3.2. Variables Used

Our study is aimed at determining the impact of location advantages, agglomeration, information cost and environmental cost effects on the localization choice of foreign pharmaceutical firms’ in China.

As explanatory variables we have used sixteen different variables related to the categorization previously discussed (Table A1). The analysis is performed at the county level. We have identified which are the counties that host foreign pharmaceutical enterprises and aggregated at the county level the total pharmaceutical FDI assets in the years 2004 and 2013. Then, we have formulated four different dependent variables to be tested (Table A1).

For the location advantage, we selected seven variables to measure its effects on the localization and investment decisions of foreign pharmaceutical enterprises. Firstly, we use four variables to estimate market factors. *gdpgrowth* is the growth rate of GDP at the province level and it is used to estimate the effects of local economic performances on FDI attraction. In order to control for the potential distortions on the coefficients due to the faster growth rates of the backward provinces, we combine the previous variable with *gdppc*, i.e., the GDP per capita at provincial level. As wealthier and highly performing economies are generally associated with higher returns, we expect that the impact of the two variables on the location selection should be positive. *market_ind* consists of the Marketization index value—an indicator, firstly proposed by Fan, Wang and Zhang [40], intended to estimate the degree of marketization reforms at the provincial level. It provides a relatively objective way to measure marketization and is widely used in the academic literature.

In our paper, we expect that the counties located in provinces characterized by a higher marketization index will be more likely to be selected as a destination for a foreign investment. *srdexp* corresponds to the amount of R&D expenditure at the provincial level that indicates the local degree of support for science and technology. Dunning [20] implies that R&D is a crucial factor influencing location decision. In our framework, as the pharmaceutical industry is highly sensitive to R&D investment, we expect that the counties situated in provinces with a higher expenditure on research and development activities will be more likely to be selected as a destination for foreign pharmaceutical enterprises. Secondly, we try to estimate the effects of trader barriers on investment location by adopting *HHI*, i.e., the Herfindahl index that is commonly used as an indicator of the regional market concentration and competitiveness at the industrial level. From this perspective, a high HHI value is associated with a monopolistic structure of the local market and to a lower degree of industrial competitiveness. According to Audretsch and Keilbach [41], market concentration reduces the opportunities for small and medium sized enterprises to purchase labor, capital or intermediate goods at lower prices. Therefore, we expect that a high HHI will negatively affect foreign firms’ location and investment decisions in a county.

In order to estimate the effects of transportation costs, we introduce *HWAY*, i.e., the length of the highway and *flightcargo*, i.e., the freight volume by air at the provincial level. As widely stressed in the literature, the configuration of an extended and efficient transport system is one of the most important preconditions for doing business activities and for FDI attraction [42,43,44]. On the other hand, it is worth considering that the latest regulation concerning GOOD MANUFACTURING PRACTICE (GMP) has invited pharmaceutical enterprises to pay more attention to product transportation modes. On this basis, we assume that transportation related factors will positively affect the location decision of pharmaceutical enterprises.

In our study, we use two variables to measure the agglomeration effect. Specifically, we adopt *gini*, i.e., the Gini coefficient of industrial location intended to measure the degree of agglomeration of the pharmaceutical sector at the provincial level. The Gini coefficient as an indicator of industrial agglomeration was first introduced by Krugman [45]. In our specification, we elaborate the Gini coefficient for industrial location on the basis of the model provided by Wen [46]. We assume that the provinces with a higher spatial Gini coefficient are more likely to appeal to foreign firms for localization and investment. In addition, we use *ratio_phar*, i.e., the pharmaceutical industry output share out of the total provincial manufacturing output to measure the local degree of sectoral specialization. We predict that the provinces with higher degrees of pharmaceutical sector value added tend to positively benefit from pharmaceutical FDI inflows.

We deal with the estimation of information cost effects by taking into account four different variables. *ratio_forind* measures the share of FDI on output at the provincial level. According to Mariotti and Piscitello [29], locating near existing FDI enterprises helps to achieve low-cost information by means of “knowledge spillover effects” due to staff turnover or business dealings. On this basis, we expect a positive effect of FDI localization. *city_lvl* is a dummy variable that informs whether the city is one of the municipalities directly under the central government, or is a city under separate state planning at the city level, thus being characterized by more direct decision-making processes, as well as well-developed fundamental infrastructure and business services. From this perspective, valuable business information is more easily collected by foreign investors. *coastcity* is a dummy variable measuring whether the city is located in the East coastal areas of the country. According to Wei et al. [47], the coastal area has long been appealing to FDI investment choices for its openness and outward orientation both from a geographic and cultural point of view [48]. *city_dev* is a dummy variable informing on whether the city has benefitted from place-based preferential policies aimed at attracting foreign investments. Several studies have analysed the positive effects of place-based policies on FDI attraction and economic performances at city and sub-regional levels [49,50].

Finally, in our research we use three variables to estimate the influences of environmental regulation on FDI localization choices. *ratio_envinvest* measures the amount of investment in environmental pollution control over the GDP at the provincial level. It is worth emphasizing that environmental regulation may consist of either qualitative policies or quantitative investment and fees. From this perspective, environmental regulation intensity has to be considered not only as a constraint but also as a factor that is likely to foster desirable outcomes, such as an improvement in environmental conditions, which in principle may address the demands of the foreign pharmaceutical firms. However, following a standard profit-maximizing firm approach, we assume that places with higher investment in environmental pollution control will be less attractive to pharmaceutical industries due to the possibility of increased costs. *river_dist* measures how distant from the nearest river is the county in which the pharmaceutical enterprises are located. *lake_dist* assesses how distant the county in which the pharmaceutical enterprises are located is from the nearest lake. According to the *Guidelines for Environmental Information Disclosure of Listed Companies* published by the Ministry of Environmental Protection in 14 September 2010, the pharmaceutical industry is categorized as one of the 16 heavy polluting industries. Within such a normative framework, enterprises should consider their own obligations to environmental protection and to meeting environmental standards, and this may influence their localization choices. Following this logic, if enterprises locate in well-regulated areas, it is more likely that they will have enough space and channels for treating waste, so they do not have an incentive to place their operations close to rivers and lakes in order to discharge sewage at a lower cost. Conversely, in the absence of environmental regulation constraints, they would be more likely to locate in proximity to rivers and lakes to exploit the advantages of cheap waste disposal. Therefore, we use these distances to proxy the stringency of environmental regulation.

### 3.3. Modelling Location Determinants and the Amount Invested

#### 3.3.1. Location Determinants

On the basis of the set of determinants identified above, we investigate pharmaceutical FDI localization, adopting two different specifications. Firstly, by using a binary probit model we estimate whether, and to what extent, the values of the explanatory variables in 2004 are likely to have influenced the FDI decision to locate in a county in 2013. Secondly, we examine whether, and to what extent, the same covariates are likely to have affected the FDI expansion in terms of total assets at the county level in the interval between 2004 and 2013.

Focusing on the location decisions of foreign pharmaceutical enterprises, we define the variable ${\mathrm{decision}}_{i,x,2013}$ that equals 1 if the enterprises decide to locate in a county *x* and 0 otherwise.

$$\begin{array}{c} {\mathrm{decision}}_{x,2013}\\ \;\;\;=\{\begin{array}{c} 1,\;when\;total\;assets\;of\;foreign\;pharaceutical\;enterprise\;in\;county\;i\;in\;2013>0\;\\ 0,\;when\;total\;assets\;of\;foreign\;pharaceutical\;enterprise\;in\;county\;i\;in\;2013=0 \end{array} \end{array}$$

Then, we estimated the model 1 as follows:

(1) $$\begin{array}{c} {\mathrm{decision}}_{i,x,2013}\\ =f({\;\mathrm{GDPgrowth}}_{x,2004},{\;\mathrm{GDPPC}}_{x,2004},\;\mathrm{market}\_{\mathrm{ind}}_{x,2004},{\mathrm{srdexp}}_{x,2004}\;{\mathrm{HHI}}_{x,2004},{\;\mathrm{HWAY}}_{x,2004},{\;\mathrm{flightcargo}}_{x,2004},\\ {\mathrm{gini}}_{x,2004},{\;\mathrm{ratiopharind}}_{x,2004},{\mathrm{ratioforind}}_{x,2004},{\mathrm{coastcity}}_{m,x,2004},{\;\mathrm{citydev}}_{m,x,2004},{\;\mathrm{citylvl}}_{m,x,2004},\\ {\;\;\;\mathrm{envirinvest}}_{x,2004},{\;\mathrm{riverdist}}_{i,x,2004},{\;\mathrm{lakedist}}_{i,x,2004}) \end{array}$$

Similarly, to examine the effects of the covariates on the changes in location, we define the variable $\Delta {\mathrm{decision}}_{i,x}$ as equal to 1 if the foreign pharmaceutical enterprise expands its size in one county, and 0 otherwise.

$$\begin{array}{c} \Delta {\mathrm{decision}}_{x}\\ \;\;=\{\begin{array}{c} 1,\;when\;the\;changes\;in\;total\;assets\;of\;those\;enterprise\;in\;county\;i\;from\;2004\;to\;2013>0\;\\ 0,\;when\;the\;changes\;in\;total\;assets\;of\;those\;enterprise\;in\;county\;i\;from\;2004\;to\;2013\leq 0\; \end{array} \end{array}$$

Therefore, we estimate the following model:

(2) $$\begin{array}{c} \Delta {\mathrm{decision}}_{i,x}\\ =f({\;\mathrm{GDPgrowth}}_{x,2004},{\;\mathrm{GDPPC}}_{x,2004},{\;\mathrm{market}}_{{\mathrm{ind}}_{x,2004}},{\mathrm{srdexp}}_{x,2004},{\;\mathrm{HHI}}_{x,2004},{\;\mathrm{HWAY}}_{x,2004},{\;\mathrm{flightcargo}}_{x,2004},\\ {\mathrm{gini}}_{x,2004},{\;\mathrm{ratiopharind}}_{x,2004},{\mathrm{ratioforind}}_{x,2004},{\mathrm{coastcity}}_{m,x,2004},{\;\mathrm{citydev}}_{m,x,2004},{\;\mathrm{citylvl}}_{m,x,2004},\\ {\;\;\;\mathrm{envirinvest}}_{x,2004},{\;\mathrm{riverdist}}_{i,x,2004},{\;\mathrm{lakedist}}_{i,x,2004}) \end{array}$$

#### 3.3.2. Amount Invested

**Figure 3:**
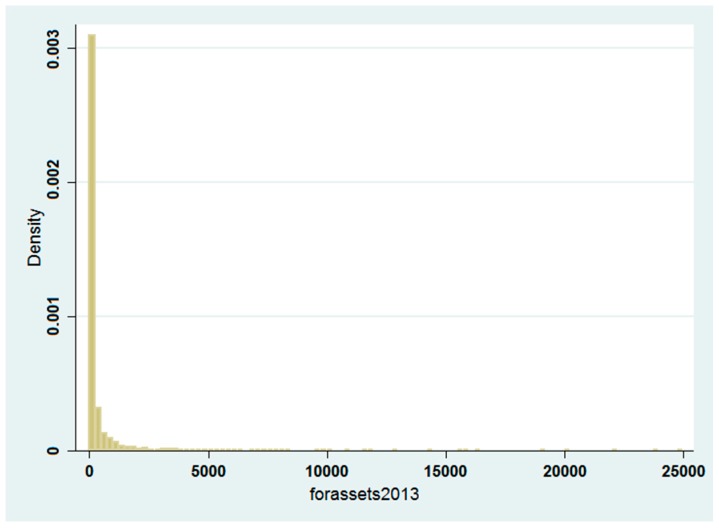
Graphic representation of the distribution of Foreign Direct Investment (FDI) total assets at county level in 2013. Source: Figure created by the Authors derived from STATA.

After analyzing the determinants of pharmaceutical FDI location decision across Chinese counties, we want to discover whether, and to what extent, our set of covariates is likely to affect the total amount of assets invested in a county, thus exploring the degree of FDI localization. As we can see from the graph in Figure 3 and the statistics in Table 1, the total amount of FDI at the county level does not follow a standard normal distribution, being concentrated around zero. Consequently, since we are in the presence of censored data, we opt to use Tobit regressions.

As before, we use two different specifications. In model 3 we estimate the effects of the covariates in 2004 on the amount of FDI total assets in the year 2013. The equation is as follows:

(3) $$\begin{array}{c} {\mathrm{forassets}}_{i,x,2013}\\ =f({\;\mathrm{GDPgrowth}}_{x,2004},{\;\mathrm{GDPPC}}_{x,2004},\;\mathrm{market}\_{\mathrm{ind}}_{x,2004},{\mathrm{srdexp}}_{x,2004}{\;\mathrm{HHI}}_{x,2004},{\;\mathrm{HWAY}}_{x,2004},{\;\mathrm{flightcargo}}_{x,2004},\\ {\mathrm{gini}}_{x,2004},{\;\mathrm{ratiopharind}}_{x,2004},{\mathrm{ratioforind}}_{x,2004},{\mathrm{coastcity}}_{m,x,2004},{\;\mathrm{citydev}}_{m,x,2004},{\;\mathrm{citylvl}}_{m,x,2004},\\ {\;\mathrm{envirinvest}}_{x,2004},{\;\mathrm{riverdist}}_{i,x,2004},{\;\mathrm{lakedist}}_{i,x,2004}) \end{array}$$

In model 4, we test the same covariates on a new dependent variable, i.e., $\Delta {\mathrm{forassets}}_{i,x}$, which corresponds to the difference between the total assets of foreign pharmaceutical enterprises in 2004 and 2013 at county level. This is for assessing to what extent the factors considered in the model are likely to have generated a change in the attraction of pharmaceutical FDI at the county level.

**Figure 4:**
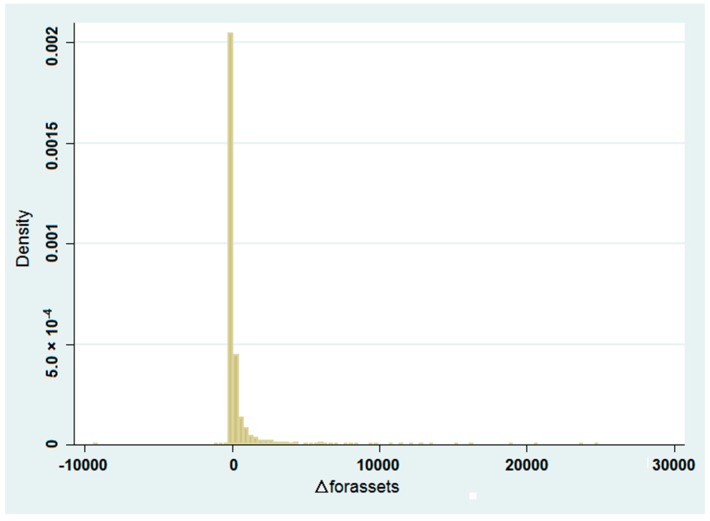
Graphic representation of the changes in FDI total assets at county level 2004–2013. Source: Figure created by the Authors derived from STATA.

As we can see from the graph in Figure 4 and the statistics in Table 2, this variable exhibits a truncated distribution, so in this case we also apply the Tobit model as follows:

(4) $$\begin{array}{c} \Delta {\mathrm{forassets}}_{i,x}\\ =f({\;\mathrm{GDPgrowth}}_{x,2004},{\;\mathrm{GDPPC}}_{x,2004},\;\mathrm{market}\_{\mathrm{ind}}_{x,2004},{\mathrm{srdexp}}_{x,2004}{\;\mathrm{HHI}}_{x,2004},{\;\mathrm{HWAY}}_{x,2004},{\;\mathrm{flightcargo}}_{x,2004},\\ {\mathrm{gini}}_{x,2004},{\;\mathrm{ratiopharind}}_{x,2004},{\mathrm{ratioforind}}_{x,2004},{\mathrm{coastcity}}_{m,x,2004},{\;\mathrm{citydev}}_{m,x,2004},{\;\mathrm{citylvl}}_{m,x,2004},\\ {\;\mathrm{envirinvest}}_{x,2004},{\;\mathrm{riverdist}}_{i,x,2004},{\;\mathrm{lakedist}}_{i,x,2004}) \end{array}$$

## 4. Results

In this section, we display and interpret the results of the four specifications that we derived previously.

In model 1 (Table 3) the data display that—among the location advantages—GDP per capita as well as higher scores in the marketization index are significantly related to pharmaceutical FDI localization at the county level. The negative coefficient on HHI is consistent with our expectations, showing that trade barriers and a monopolistic market structure is likely to prevent entry for foreign firms. However, expenditure on R&D seems not to have a significant role in determining pharmaceutical FDI localization choices. Looking at the influence of the local transportation endowments on FDI attraction, the data show a positive but not significant effect of the length of highway and a negative coefficient for the freight volume by airline.

Focusing on the agglomeration advantage effects, it is possible to notice that the Gini coefficient of industrial location, although positive, is not significant. At the same time, the data provide evidence suggesting that a higher share of pharmaceutical enterprises in the provincial industrial structure is likely to attract more FDI operating in the same sector.

Analysing the effects of the variables linked to the information cost, it is worth mentioning that, in contrast with our expectations, a local foreign presence is likely to negatively affect pharmaceutical FDI localization decisions. This may be due to saturation and over-competition dynamics that generally discourage new entrants, pushing them towards less competitive markets. In addition, coastal areas also report negative coefficients suggesting the negative effect on FDI attraction caused by the progressive reduction of local support policies based on a FDI-led model of industrialization and the simultaneous promotion of FDI attraction policies in the internal areas. Conversely, the presence of municipalities under separate state-planning as well as of cities implementing preferential policies tends to generate positive effects on FDI localization.

Finally, with respect to the cost of environmental regulation, it is interesting to find that those provinces investing more money in environmental protection are less likely to be attractive for pharmaceutical FDI location.

Considering model 2 (Table A4), relating to the pharmaceutical FDI expansion in the period between 2004 and 2013, we get similar results both in terms of signs and significance. The only relevant difference concerns the significant and positive effect of highway length on FDI growth at the county level. This is reasonable from a dynamic perspective, especially if we consider the consistent impact of transportation endowments on the FDI attraction.

Analysing the results from model 3, related to the Tobit regression on the amounts invested by the foreign enterprises at the county level, the data still report similar evidence (Table 4). In this regard, the only two differences are: firstly, the positive coefficient on R&D expenditure highlighting a better responsiveness of foreign firms’ degree of investment with respect to those counties characterized by higher innovative endowments—from this perspective it is important to notice that the use and the process of data concerning foreign firms invested amounts provide additional information compared to the mere localization decision—secondly, the two coefficients relating to the distance from the river and lake exhibit significant coefficients but with opposite signs, respectively positive and negative. On this basis, the proximity to a river is likely to be associated with lower amounts of pharmaceutical foreign investment, while the closer the county is to a lake, the greater is its probability of attracting higher amounts of pharmaceutical FDI. Finally, model 4—relating to the changes in the amount of FDI in the interval between 2004 and 2013—confirms the results of the first Tobit specification both in terms of signs and significance of the coefficients (Table A5).

## 5. Conclusions

Direct investments have been widely recognized as a crucial feature of the Chinese industrial development process. However, the Chinese market exhibits large dissimilarities in terms of FDI localization across territories. We choose to analyze the pharmaceutical industry as one of the key priority sectors selected by the Chinese government both to support the industrial enhancement of China towards a more R&D oriented country, and to feed the internal booming demand for healthcare services and products. Our study examines the determinants of pharmaceutical FDI localization across Chinese counties. It focuses both on the firms’ localization decisions and their invested amounts in a specific location. To draw out a more systematic framework about the pharmaceutical FDI localization dynamics, we build an augmented version of Dunning’s OLI model that takes into account four different categories of determinants such as location advantages, agglomeration cost, information cost and environmental cost effects.

We find that pharmaceutical FDI localization is positively affected by factors related to the location advantage such as GDP level and the degree of local marketization, while it is negatively associated with lower degrees of competition in the market structure. As for the agglomeration advantages, the data do exhibit significant effects related to the positive impact of local industrial specialization in the pharmaceuticals on FDI localization trends.

Focusing on information cost effects, in contrast with conclusions drawn in the prevalent literature, our findings suggest that a higher presence of FDI at the local level is likely to discourage foreign pharmaceutical firms’ from deciding to locate there. This may be due to over-competition and market congestion dynamics affecting the operation of new entrants in the pharmaceutical sector and pushing them towards less competitive markets. At the same time, the promotion of preferential policies and other place-based measures seems to stimulate a positive impact on FDI localization at the county level, while the geographical variable related to location in coastal areas does not provide explanatory power.

Finally, examining the effect of environmental costs, it is worth highlighting that those counties located in provinces investing more money in environmental control are less likely to be attractive for pharmaceutical FDI location. This suggests an adverse inclination of the investing firms towards environmental protection issues. The aforementioned results are confirmed even considering total pharmaceutical FDI assets as a dependent variable, with the only exception of local R&D expenditure, which becomes significant and positively associated with the amount invested.

Such findings are quite relevant for policymakers. The study demonstrates the heterogeneity of China in terms of its capacity to attract foreign firms, based not only on local resources and endowments but also on differentiated local systems of incentives, which might influence location choice and affect the decisions of the international ventures. From this perspective, it is clear that location choices are significantly affected by selective policies promoted at territorial level.

The availability of incentives is a key determinant in the location choice of foreign investors. The same logic also applies to the possibility of avoiding the stringent environmental regulation experienced in their home countries. The last one is a central issue in the light of orientation, from the Chinese side, of future policies to continue attracting foreign investors and promoting learning spillovers. Considering that China is strongly committed to a green environment and is at the front line in fighting against climate change, it is hard to imagine that relaxed environmental standards will be used as a way to attract foreign investments.

From a Western perspective it appears clear that, given the vast heterogeneity of the Chinese market, the promotion policies to invest abroad should not be tailored at the country-level, and should focus instead on a differentiation of the targets based on local specificities.

Despite the novelty of our study, we can identify some limitations and further avenues for research. There is the need to improve the quantitative model by including more variables for detecting determinants of FDI at a more disaggregated level than Chinese provinces, including more proxies of R&D capacity and industry specificity. Also, firm-level qualitative analysis via questionnaire or direct interview could help in better understanding the determinants of location choice.

## Figures and Tables

**Figure 2:**
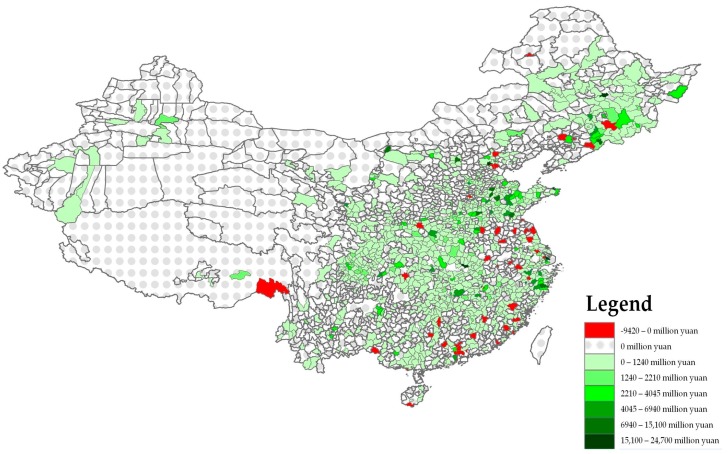
Changes in total assets of foreign pharmaceutical enterprises 2004–2013. Source: Figure created by the Authors derived from Arcgis.

**Table 1:** Basic statistics about the distribution of FDI total assets at county level in 2013.

Variable	Observation	Mean	Max	Min	Median	SD
${\mathrm{forassets}}_{i,x,2013}$	2766	410.0457	24,781.16	0	0	1514.62

**Table 2:** Basic statistics about the changes in FDI total assets at county level 2004–2013.

Variable	Observation	Mean	Max	Min	Median	SD
$\Delta {\mathrm{forassets}}_{i,x}$	2676	367.3223	24,651.82	−9411.238	0	1425.901

**Table 3:** The determinants of foreign pharmaceutical enterprise localization.

		Probit2013 _Location Advantage	Probit2013 _Agglomeration Effect	Probit2013 _Information Cost	Probit2013
Location advantage	gdpgrowth	0.014 *	0.032 ***	0.014	0.014
		(0.008)	(0.010)	(0.011)	(0.011)
	gdppc	0.052 ***	0.032 ***	0.042 ***	0.049 ***
		(0.011)	(0.011)	(0.011)	(0.012)
	srdexp	0.067	0.018	−0.001	0.063
		(0.069)	(0.069)	(0.071)	(0.074)
	HHI	−4.139 ***	−4.126 ***	−4.530 ***	−3.511 ***
		(0.620)	(0.665)	(0.655)	(0.686)
	market_ind	−0.116 ***	0.022	0.108 ***	0.121 ***
		(0.036)	(0.038)	(0.041)	(0.042)
	HWAY	0.618 *	−0.570	−0.303	0.180
		(0.359)	(0.403)	(0.403)	(0.421)
	flightcargo	−0.516 ***	−0.284 **	−0.418 ***	−0.542 ***
		(0.149)	(0.144)	(0.147)	(0.154)
Agglomeration effect	gini		−0.117	0.219	0.257
			(0.269)	(0.286)	(0.286)
	ratio_phar		0.163 ***	0.159 ***	0.130 ***
			(0.017)	(0.017)	(0.018)
Information cost	ratio_forind			−0.017 ***	−0.019 ***
				(0.003)	(0.003)
	coastcity			−0.154 *	−0.175 **
				(0.089)	(0.089)
	city_dev			0.281 ***	0.300 ***
				(0.057)	(0.058)
	city_lvl			0.251	0.269 *
				(0.154)	(0.154)
Environment Regulation	ratio_envinvest				−0.306 ***
					(0.097)
	river_dist				0.000
					(0.000)
	lake_dist				−0.003
					(0.002)
	_cons	0.105	−1.125 ***	−1.241 ***	−1.050 **
		(0.269)	(0.403)	(0.403)	(0.427)

Standard errors in brackets. Significance levels: *** *p* < 0.01; ** *p* < 0.05; * *p* < 0.1.

**Table 4:** The determinants of foreign pharmaceutical firms invested amount.

		Tobit2013_Location Advantage	Tobit2013_Agglomeration Effect	Tobit2013_Information Cost	Tobit2013
Location advantage	gdpgrowth	84.841 ***	74.177 ***	36.385	41.950 *
		(18.715)	(23.223)	(23.624)	(23.815)
	gdppc	100.690 ***	106.101 ***	115.950 ***	127.814 ***
		(22.306)	(22.162)	(23.019)	(23.629)
	srdexp	355.556 **	215.123	221.050	312.992 **
		(146.780)	(147.555)	(149.708)	(155.223)
	HHI	−7665.700 ***	−8040.319 ***	−8985.053 ***	−6770.571 ***
		(1315.367)	(1332.224)	(1314.919)	(1423.379)
	market_ind	−53.011	−3.690	147.964 *	191.030 **
		(79.934)	(80.201)	(84.245)	(85.156)
	HWAY	1299.916	−314.149	477.298	1409.809
		(799.608)	(856.299)	(848.447)	(899.291)
	flightcargo	−544.472 *	−442.756	−617.843 **	−804.071 ***
		(299.078)	(299.022)	(295.979)	(307.559)
Agglomeration effect	gini		−696.175	−415.323	−152.350
			(611.770)	(632.488)	(635.149)
	ratio_phar		249.177 ***	217.827 ***	174.721 ***
			(38.716)	(38.262)	(41.223)
Information cost	ratio_forind			−32.137 ***	−37.400 ***
				(6.043)	(6.215)
	coastcity			−315.659 *	−355.009 *
				(185.733)	(185.666)
	city_dev			1009.440 ***	1037.198 ***
				(118.421)	(118.881)
	city_lvl			291.929	290.116
				(299.745)	(300.517)
Environment Regulation	ratio_envinvest				−580.630 ***
					(177.848)
	river_dist				1.043 **
					(0.451)
	lake_dist				−10.203 **
					(5.018)
	_cons	−3028.162 ***	−3207.375 ***	−3103.931 ***	−3177.655 ***
		(636.321)	(904.430)	(895.603)	(909.696)
	/sigma	2367.942 ***			
		(48.814)			
	/sigma		2359.971 ***		
			(48.596)		
	/sigma			2310.534 ***	
				(47.378)	
	/sigma				2304.973 ***
					(47.250)

Standard errors in brackets. Significance levels: *** *p* < 0.01; ** *p* < 0.05; * *p* < 0.1.

## Appendix A

**Table A1:** Variables and Data Sources.

**Dependent Variables**		**Measurement**	**Data Source**	
	decision	Decide to locate in one county or not	China’s industrial Enterprise Database	model (3)
	∆decision	Changes in the decision on whether to locate in one county or not	China’s industrial Enterprise Database	model (4)
	forassets	Total assets of FDI pharmaceutical enterprises at the county level (million yuan)	China’s industrial Enterprise Database	model (5)
	∆forassets	Changes in total assets of FDI pharmaceutical enterprises at the county level (million yuan)	China’s industrial Enterprise Database	model (6)
**Independent Variables**		**Measurement**	**Data Source**	**Expected Symbol**
Location Advantage	gdpgrowth	The growth rate of GDP at provincial level (%)	China Statistical Yearbook	+
	gdppc	The GDP per at provincial level (thousand yuan per capita)	China Statistical Yearbook	+
	market_ind	The Marketization Index at the provincial level	Fan et al. (2000)	+
	rdexp	The expenditure on R&D over GDP at provincial level (million yuan per 100 million yuan)	China Statistical Yearbook	+
	HHI *	The Herfindahl index at provincial level	China’s industrial Enterprise Database	-
	HWAY	The highway length over the GDP at provincial level (km per 100 million yuan)	China Statistical Yearbook	+
	flightcargo	The total amount of the freight volume by air at provincial level (million ton per 100 million yuan)	China Statistical Yearbook	+
Agglomeration Effect	gini *	Gini coefficient of industrial location at provincial level	China’s industrial Enterprise Database	+
	ratio_phaind	Share of pharmaceutical industry over the total industrial output at provincial level (%)	China’s industrial Enterprise Database	+
Information Cost	ratio_forind	Share of industrial output of foreign enterprises at provincial level (%)	China’s industrial Enterprise Database	+
	coastcity	1 = if a city locates in coastal area on city level; 0 = others	China Statistical Yearbook	+
	city_dev	1 = if a city is special economic zones, high-tech industrial zones on city level, and bonded zone ; 0 = others	Government website	+
	city_lvl	1 = if a city is one of municipalities directly under the central government, or cities under separate state planning on city level; 0 = others	China Statistical Yearbook	+
Environment Regulation	ratio_envinvest	The share of environment investment in pollution treatment over the GDP at provincial level (%)	China Statistical Yearbook	-
	river_dist	The distance of the location county from the nearest river (km)	Arcgis	+
	lake_dist	The distance of the location county from the nearest lake (km)	Arcgis	+

* The construction of the HHI and the Gini coefficient of Industrial location is derived in Appendix B.

**Table A2:** The Top 5 Counties for the total assets of foreign pharmaceutical enterprises in 2013.

NO.	County	City	Province	Total Assets of Foreign Pharmaceutical Enterprises in 2013
1	Huancui District	Weihai	Shandong	24,781.16
2	Yuhua District	Shijiangzhuang	Hebei	23,864.41
3	Daxing District	Beijing	Beijing	22,213.68
4	Pudong New Area	Shanghai	Shanghai	20,185.4
5	Daoli District	Harbin	Heilongjiang	19,064.39

Source: China’s industrial Enterprise Database.

**Table A3:** The Top 5 Counties for changes in total assets of foreign pharmaceutical enterprises.

NO.	County	City	Province	Changes in Total Assets of Foreign Pharmaceutical Enterprises
1	Huancui District	Weihai	Shandong	24,651.82
2	Yuhua District	Shijiazhuang	Hebei	23,864.41
3	Daxing District	Beijing	Beijing	20,719.61
4	Daoli District	Harbin	Heilongjiang	19,064.39
5	Hongshan District	Wuhan	Hubei	16,278.2

Source: China’s industrial Enterprise Database.

**Table A4:** The determinants of the changes in pharmaceutical FDI localization (2004–2013).

		Probit0413_Location Advantage	Probit0413_Agglomeration Effect	Probit0413_Information Cost	Probit0413
Location Advantage	gdpgrowth	0.010	0.031 ***	0.012	0.011
		(0.008)	(0.010)	(0.011)	(0.011)
	gdppc	0.045 ***	0.027 ***	0.040 ***	0.046 ***
		(0.010)	(0.010)	(0.011)	(0.011)
	srdexp	0.052	0.005	−0.010	0.043
		(0.067)	(0.067)	(0.070)	(0.072)
	HHI	−4.139 ***	−4.109 ***	−4.482 ***	−3.618 ***
		(0.591)	(0.605)	(0.611)	(0.668)
	market_ind	−0.119 ***	0.007	0.096 **	0.108 ***
		(0.033)	(0.035)	(0.038)	(0.039)
	HWAY	1.174 ***	0.154	0.437	0.850 **
		(0.331)	(0.358)	(0.365)	(0.392)
	flightcargo	−0.639 ***	−0.439 ***	−0.564 ***	−0.658 ***
		(0.139)	(0.141)	(0.143)	(0.148)
Agglomeration Effect	gini		0.051	0.408	0.428
			(0.271)	(0.284)	(0.284)
	ratio_phar		0.153 ***	0.148 ***	0.124 ***
			(0.014)	(0.014)	(0.016)
Information Cost	ratio_forind			−0.017 ***	−0.020 ***
				(0.003)	(0.003)
	coastcity			−0.168 *	−0.187 **
				(0.090)	(0.090)
	city_dev			0.223 ***	0.240 ***
				(0.056)	(0.057)
	city_lvl			0.171	0.186
				(0.153)	(0.153)
Environment Regulation	ratio_envinvest				−0.234 ***
					(0.080)
	river_dist				−0.000
					(0.000)
	lake_dist				−0.005 **
					(0.002)
	_cons	0.276	−1.022 **	−1.143 ***	−0.989 **
		(0.261)	(0.398)	(0.403)	(0.412)

Standard errors in brackets. Significance levels: *** *p* < 0.01; ** *p* < 0.05; * *p* < 0.1.

**Table A5:** The determinants of the changes in pharmaceutical FDI invested amount (2004–2013).

		Tobit0413_Location Advantage	Tobit0413_Agglomeration Effect	Tobit0413_Information Cost	Tobit0413
location Advantage	gdpgrowth	83.919 ***	64.916 ***	25.160	31.150
		(18.144)	(22.703)	(23.084)	(23.259)
	gdppc	79.603 ***	83.733 ***	100.285 ***	107.792 ***
		(21.821)	(21.654)	(22.629)	(23.268)
	srdexp	326.456 **	186.433	214.047	278.015 *
		(141.967)	(142.562)	(144.669)	(150.233)
	HHI	−7149.947 ***	−7498.719 ***	−8248.028 ***	−6342.834 ***
		(1278.475)	(1292.231)	(1275.198)	(1377.545)
	market_ind	−43.429	0.734	167.314 **	209.628 **
		(78.357)	(78.587)	(82.763)	(83.664)
	HWAY	1100.598	−566.094	369.787	1084.803
		(817.245)	(870.649)	(861.233)	(913.112)
	flightcargo	−599.471 **	−470.214	−588.915 **	−716.102 **
		(296.621)	(295.810)	(293.695)	(305.505)
Agglomeration Effect	gini		−1045.692*	−715.338	−485.865
			(599.660)	(618.721)	(621.509)
	ratio_phar		239.193 ***	208.480 ***	176.476 ***
			(37.015)	(36.614)	(39.576)
Information Cost	ratio_forind			−34.302 ***	−38.535 ***
				(5.869)	(6.031)
	coastcity			−333.173 *	−365.243 **
				(180.769)	(180.665)
	city_dev			894.631 ***	915.165 ***
				(114.979)	(115.429)
	city_lvl			−17.181	−46.279
				(302.799)	(303.564)
Environment Regulation	ratio_envinvest				−459.468 ***
					(171.277)
	river_dist				1.202 ***
					(0.436)
	lake_dist				−10.570 **
					(4.910)
	_cons	−2830.819 ***	−2600.085 ***	−2593.192 ***	−2762.395 ***
		(621.130)	(890.612)	(883.218)	(895.809)
	/sigma	2248.061 ***			
		(47.675)			
	/sigma		2238.403 ***		
			(47.402)		
	/sigma			2194.646 ***	
				(46.297)	
	/sigma				2189.371 ***
					(46.172)

Standard errors in brackets. Significance levels: *** *p* < 0.01; ** *p* < 0.05; * *p* < 0.1.

## Appendix B

**1. HHI**

HHI is the quadratic sum of market share of industry *i* in province *x* as below.

(A1) $$H=\underset{i}{\sum }S_{i}^{2}$$

where $S_{i}$ is the market share of industry *i*, where we use the proportion of industrial output of pharmaceutical enterprises to measure market share in one province.

**2. Gini Coefficient for Industry**

Wen (2004) calculates the Gini coefficient for industry in 1980, 1985 and 1995 in China. The formula is as below.

(A2) $$G^{i}=\frac{1}{2n^{2}\bar{S^{i}}}\overset{n}{\underset{k=1}{\sum }}\overset{n}{\underset{j=1}{\sum }}|\;s_{j}^{i}-s_{k}^{i}\;|$$

where $s_{j}^{i}$ is the market share of industry i in province *j*, and $s_{k}^{i}$ is the market share of industry *i* in province *k*, *n* is the number of provinces, and $\bar{S^{i}}$ is the average market share of industry *i* in the region. In our research, we need to calculate the Gini coefficient for pharmaceutical industry in each province. Therefore, we add the provincial dimension *x* in the formula.

(A3) $$G_{x}^{i}=\frac{1}{2{n_{x}}^{2}\bar{s_{x}^{i}}}\overset{n}{\underset{k=1}{\sum }}\overset{n}{\underset{j=1}{\sum }}|\;s_{jx}^{i}-s_{kx}^{i}\;|$$

In Formula (A3), we estimate the gini coefficient for pharmaceutical industry in province *x*, where $s_{jx}^{i}$ is the market share of industry *i* in city *j* of province *x*, and $s_{kx}^{i}\;$is the market share of industry *i* in city *k* in province *x*, *n* is the number of city in province *x*, and $\bar{s_{x}^{i}}$ is the average market share of industry *i* in province *x*. We use the aggregate industrial output of enterprises to the city level to estimate the market share of pharmaceutical industry.
